# The effect of simulated toothbrushing with regular and whitening dentifrices on the colour stability of high translucency zirconia

**DOI:** 10.2340/biid.v13.46463

**Published:** 2026-08-06

**Authors:** Basapogu Sreeramulu, Varsha Umesh Revankar, Akula Vineela Rani, Anusha Reddy Pucha

**Affiliations:** Department of Prosthodontics, Government Dental College and Hospital, Hyderabad, Telangana, India

**Keywords:** High translucency zirconia, colour stability, toothbrushing simulation, dentifrice abrasion, CIELAB, spectrophotometry

## Abstract

**Background:**

High translucency zirconia (HTZ) restorations are increasingly used for aesthetic dental applications due to their superior optical properties. However, colour stability under routine oral hygiene procedures, especially toothbrushing with regular or whitening dentifrices, remains a clinical concern. This study evaluated the effect of simulated toothbrushing on the colour stability (ΔE) of two different HTZ materials.

**Methods:**

Sixty unglazed and unpolished HTZ samples (10 mm × 2 mm) were fabricated using Computer Aided Design and Computer Aided Manufacturing (CAD/CAM) technology and divided into two groups based on translucency (HTZ-A: 5.8–9.7 wt% yttria and HTZ-B: 4.4–5.5 wt% yttria; *n* = 30 each). Each group was subdivided into three subgroups (*n* = 10) according to the treatment: artificial saliva (control), regular dentifrice and whitening dentifrice. Baseline colour measurements were recorded using a reflectance spectrophotometer. Specimens underwent simulated toothbrushing or storage in artificial saliva for 520 min, simulating approximately 17 years of brushing. Colour differences (ΔE) were calculated using the CIELAB system. Data were analysed using one-way Analysis of Variance (ANOVA), Tukey’s post-hoc test and independent t-tests.

**Results:**

Artificial saliva caused minimal colour change in both zirconia groups (ΔE = 1.677 for HTZA, 1.802 for HTZB). Regular dentifrice induced significant ΔE values (HTZ-A: 8.722, HTZ-B: 8.346), while whitening dentifrice caused a significantly highest colour change for HTZ-B (HTZ-A: 6.348, HTZ-B: 11.062). Pairwise comparison revealed significant differences among all subgroups (*p* < 0.05). Intergroup analysis showed a significant difference between HTZ-A and HTZ-B only in the whitening dentifrice subgroup (*p* = 0.0001).

**Conclusion:**

Simulated toothbrushing significantly affects the colour stability of HTZ restorations. Whitening dentifrices produced the greatest colour change in highly translucent zirconia. Material composition and dentifrice selection should be considered to maintain long-term aesthetics of zirconia restorations.

## Introduction

Aesthetics in dentistry encompasses the principles of beauty and visual harmony, aiming to recreate natural tooth appearance while restoring functional integrity [1]. Contemporary advances in dental materials and fabrication techniques have significantly improved the aesthetic potential of ceramic restorations. Despite these developments, maintaining long-term aesthetic stability remains a critical clinical challenge. Among the parameters influencing clinical success, colour stability is considered one of the most important determinants of longevity in aesthetic prostheses. Colour difference in dental materials is commonly quantified using the CIELAB colour system, where the overall colour change is expressed as ΔE. To interpret the clinical relevance of these values, perceptibility and acceptability thresholds have been proposed. A ΔE value of approximately 0.8 represents the perceptibility threshold, indicating the smallest colour difference detectable by the human eye under controlled conditions, whereas a ΔE value of approximately 1.2 represents the clinical acceptability threshold, beyond which the colour difference is generally considered unacceptable in clinical dentistry. These thresholds provide a useful reference for evaluating the colour stability of restorative materials.

Several mechanisms may contribute to discoloration of restorative materials in the oral environment, including: (1) extrinsic staining from plaque and surface pigments, (2) intrinsic discoloration due to material aging and (3) surface alteration caused by degradation or penetration of staining agents [2]. Toothbrushing, the most widely practiced oral-hygiene measure, represents a three-body abrasion process, in which toothbrush bristles and dentifrice abrasives interact with the restorative surface [3]. The chemical composition and abrasivity of dentifrices-measured by the relative dentin abrasion (RDA) index can influence surface roughness, optical properties and plaque-retention potential [4]. High fluoride and whitening dentifrices have been previously shown to modify the surface integrity and optical behaviour of dental ceramics [5, 6].

Zirconia-based ceramics have gained prominence due to their mechanical strength, biocompatibility and aesthetic potential. Zirconia exists in monoclinic, tetragonal and cubic crystal phases, with phase stability governed by temperature and stabilising oxides [7]. Zirconia materials are commonly classified according to their yttria stabiliser content expressed in molar percentage (mol%), such as 3Y-TZP, 4Y-PSZ and 5Y-PSZ. However, manufacturers often report composition in weight percentage (wt%). Conventional 3 mol% yttria-stabilised tetragonal zirconia polycrystal (3Y-TZP) offers high strength but limited translucency. To address this, increased yttria concentrations (4–5 mol%) have been used to stabilise greater proportions of the cubic phase, yielding 4 mol% yttria (4.4–5.5 wt% yttria) partially stabilised zirconia (4Y-PSZ) and 5 mol% yttria (5.8–9.7 wt% yttria) partially stabilised zirconia (5Y-PSZ) with enhanced translucency [8]. These materials allow fabrications with superior light transmission and improved optical outcomes, aligning with current aesthetic demands.

Given the increasing clinical use of high translucency zirconia (HTZ) and the widespread daily practice of toothbrushing, it is essential to determine whether toothbrushing with various dentifrices affects the colour stability of these advanced ceramic materials. Therefore, the present study aimed to investigate the effect of simulated toothbrushing on the colour stability of two HTZ systems. The null hypothesis stated that there would be no difference in colour change between the two HTZs and there would be no significant effect of dentifrice on the colour change of the two HTZs.

## Materials and methods

### Specimen preparation

A total of 60 unglazed and unpolished HTZ specimens (Cera Motion Z Blank Cubic Multishade: Dentaurum GmbH & Co.KG, Ispringen, Germany) were fabricated for this study. Each specimen was standardised to a diameter of 10 ± 0.1 mm and a thickness of 2 ± 0.1 mm. The specimen design was created using computer-aided design (CAD) software, after which the digital design was transferred to a CAD/CAM milling machine for subtractive fabrication from pre-sintered zirconia blanks. The specimens were intentionally left unglazed and unpolished to standardise the surface condition and avoid the influence of surface finishing procedures on colour stability. Surface treatments such as glazing and polishing can alter surface roughness and optical properties, potentially confounding the evaluation of colour changes caused specifically by dentifrice abrasion. Moreover, in clinical situations zirconia restorations often undergo chairside occlusal adjustments after placement, which may remove the glaze layer and expose the underlying zirconia surface that is subsequently polished. Therefore, the use of unglazed specimens allowed assessment of the intrinsic material response to simulated toothbrushing under controlled conditions. The 60 specimens were then divided into two major groups (*n* = 30 per group) according to material translucency based on yttria content. Each major group was subsequently divided into three subgroups (*n* = 10 per subgroup) based on the brushing procedure and dentifrice used (Table 1).

**Table 1:** Grouping of high translucency zirconia samples based on dentifrice exposure.

Major group	Material type	Subgroup	Treatment condition	*N*
**A**	High-translucency Zirconia-A (5.8–9.7 wt% yttria)	Aa	Artificial saliva	10
		Ab	Regular dentifrice	10
		Ac	Whitening dentifrice	10
**B**	High-translucency Zirconia-B (4.4–5.5 wt% yttria)	Ba	Artificial saliva	10
		Bb	Regular dentifrice	10
		Bc	Whitening dentifrice	10

### Baseline colour measurement

Prior to storage or simulated toothbrushing, all specimens were subjected to ultrasonic cleaning in distilled water for 5 min to remove surface contaminants. Baseline colour measurements were then recorded for all specimens.

Colour evaluation was performed using a reflectance spectrophotometer (Cary 60 UV-Vis Reflectance Spectrophotometer [Agilent Technologies India Private Ltd., Hyderabad, India]) equipped with a diffuse reflectance accessory under standardised lighting conditions. Each specimen was positioned on a neutral grey background, and three consecutive measurements were taken at the centre of the surface. The mean L*, a* and b* values were recorded as the baseline colour coordinates for each specimen.

### Preparation of artificial saliva (control solution)

Artificial saliva was prepared according to the standard formulation using the following components dissolved in 1,000 mL of deionised water [9]:

- 1.21 g Potassium chloride (KCl)
- 0.40 g Sodium chloride (NaCl)
- 0.005 g Sodium sulphide nonahydrate (Na_2_S·9H_2_O)
- 1.0 g Urea [CO(NH_2_)_2_]
- 0.78 g Sodium dihydrogen phosphate dihydrate (NaH_2_PO_4_·2H_2_O)

The pH was adjusted to 7.02 ± 0.15 using 10N Sodium hydroxide. A total of 20 specimens (10 from each major group) representing the control subgroups (Aa and Ba) were fully submerged in artificial saliva at 37°C in an incubator for 520 min to simulate intraoral conditions during the experimental period. This storage duration corresponded to the brushing period applied in the experimental groups.

### Preparation of dentifrice slurries

Two dentifrices – regular dentifrice (Colgate Total, Colgate Palmolive Company Ltd., Mumbai, India) and whitening dentifrice (Colgate Optic White, Colgate Palmolive Company Ltd., Mumbai, India) – were used. Each dentifrice was mixed with artificial saliva in a ratio of 1:4 (w/v) to prepare slurries in accordance with ISO 11609:2017 standards for toothpaste testing. Fresh slurry was prepared at the start of each brushing cycle.

### Simulated toothbrushing procedure

**Figure 1:**
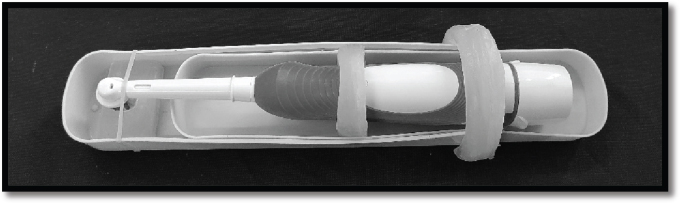
Customised tooth brush holding device.

Simulated toothbrushing was performed using a battery-powered toothbrush (Precision Clean; Oral-B Braun, Procter & Gamble, Germany) fitted with a cup-shaped brush head mounted onto a customised brushing apparatus designed to stabilise movement and maintain uniform brush contact (Figure 1). A constant vertical load of 2N was applied to each specimen during brushing [10, 11]. The force was standardised using 0.5-inch orthodontic extraoral elastics (Penta Ortho Extraoral Elastics, Penta Orthodontics, Achanpady, Kerala, India), ensuring reproducibility across all samples.

The total brushing time was calculated based on the brushing time of 120 s two times a day for all 28 teeth. As a tooth has several surfaces to be brushed, the maximum contact time per tooth surface per day has been reported to be 5 s [12, 13]. In addition, the simulated brushing time of 520 min for one surface of the sample was evaluated to be equivalent to 17 years of tooth brushing [12, 13]. The samples in the control group remained submerged in the artificial saliva for 520 min.

### Post-brushing cleaning and colour measurement

Following simulated brushing, specimens were rinsed under tap water for 30 s to remove residual dentifrice slurry. Colour measurements were repeated using the same spectrophotometric protocol described for baseline evaluation. Three readings were obtained per specimen, and the mean L*, a* and b* values were used to calculate colour change (ΔE) using the CIELAB 2000 (ΔE₀₀) formula, which accounts for perceptual non-uniformities in lightness, chroma and hue. ΔE₀₀ provides a more accurate reflection of human visual perception than ΔE*ab (1976) and is widely recommended for evaluating clinically relevant colour changes in dental materials:


$$E=\sqrt{{\left(L^{*}\right)}^{2}+{\left(a^{*}\right)}^{2}+{\left(b^{*}\right)}^{2}}$$

where ΔL*, Δa* and Δb* represent differences between baseline and post-brushing values.

### Statistical analysis

The colour change values (ΔE*) were subjected to statistical analysis using IBM SPSS Statistics (Version 20.0, Armonk, NY). Prior to inferential analysis, the normality of data distribution was assessed using the Shapiro–Wilk test, and Levene’s test was used to evaluate homogeneity of variance. As the assumptions for parametric testing were satisfied, one-way Analysis of Variance (ANOVA) followed by Tukey’s post-hoc test was applied for mean comparison of ΔE values among subgroups within each major group. An independent samples t-test was used to compare ΔE values between the two major zirconia groups. A *p*-value ≤ 0.05 was considered statistically significant.

## Results

The mean colour change (ΔE) of HTZ-A varied between 1.677 and 8.722. The one-way ANOVA revealed a statistically significant difference among the three subgroups (*F* = 174.5, *p* = 0.001). Subsequently, Tukey’s post-hoc tests found statistically significant differences in ΔE for all pairwise comparisons of subgroups (*p* = 0.0001). Thus, both regular (Ab) and whitening dentifrices (Ac) caused significantly greater colour change than artificial saliva (Aa), and regular dentifrice induced more colour change than whitening dentifrice (Tables 2 and 3).

**Table 2:** Mean comparison of ΔE between subgroups of HTZ–A and HTZ-B samples using Tukey’s post-hoc test.

Major group	Subgroup comparing	Subgroup compared	Mean difference	*P*
HTZ-A	Artificial saliva (Aa)	Regular dentifrice (Ab)	-7.04463*	0.0001
		Whitening dentifrice (Ac)	-4.67150*	0.0001
	Regular dentifrice (Ab)	Whitening dentifrice (Ac)	2.37312*	0.0001
HTZ-B	Artificial saliva (Ba)	Regular dentifrice (Bb)	-6.54400*	0.0001
		Whitening dentifrice (Bc)	-9.26012*	0.0001
	Regular dentifrice (Bb)	Whitening dentifrice (Bc)	-2.71613*	0.0001

HTZ-A: Cera Motion Z Blank Cubic Multishade, Dentaurum GmbH & Co.KG, Ispringen, Germany.

HTZ-B: Cera Motion Z HT Shade, Dentaurum GmbH & Co.KG, Ispringen, Germany.

* differentiates the CIE standard from Hunter’s Lab, an earlier color model developed in the 1940s, which also used L, a, and b but was not formally standardized

**Table 3:** Mean comparison of ΔE among subgroups of HTZ–A and HTZ-B samples using one-way ANOVA test.

Major group	Sub-group	Mean ΔE	SD	*F*	*P*
HTZ-A	Artificial saliva (Aa)	1.6774	0.23322	174.5	0.001
	Regular dentifrice (Ab)	8.7220	0.39434		
	Whitening dentifrice (Ac)	6.3489	1.24801		
HTZ-B	Artificial saliva (Ba)	1.8023	0.80297	325.2	0.001
	Regular dentifrice (Bb)	8.3463	0.93662		
	Whitening dentifrice (Bc)	11.0624	0.38726		

SD: standard deviation; HTZ-A: Cera Motion Z Blank Cubic Multishade, Dentaurum GmbH & Co.KG, Ispringen, Germany; HTZ-B: Cera Motion Z HT Shade, Dentaurum GmbH & Co.KG, Ispringen, Germany.

The mean colour change (ΔE) of HTZ-B varied between 1.802 and 11.062. One-way ANOVA revealed a statistically significant difference among the three subgroups (*F* = 325.2, *p* = 0.001) (Tables 2 and 3). Subsequently, Tukey’s post-hoc analyses demonstrated statistically significant differences in ΔE for all pairwise comparisons of the subgroups (*p* = 0.0001). Thus, whitening dentifrice (Bc) caused the significantly highest colour change, followed by regular dentifrice (Bb), with artificial saliva (Ba) showing minimal change.

There was no significant difference in ΔE between HTZ-A and HTZ-B following brushing with artificial saliva or regular dentifrice (*p* > 0.05). In contrast, brushing with whitening dentifrice resulted in HTZ-B showing significantly higher colour change compared to HTZ-A (*p* = 0.0001). This suggests that material type influences colour stability when exposed to whitening dentifrice (Table 4).

**Table 4:** Mean comparison of ΔE between high translucency zirconia – A samples and high translucency zirconia – B samples using independent sample t-test.

Sub-group	Group	Mean	SD	SE	*P*
Artificial saliva	HTZ-A	1.677	0.233	0.082	0.679
	HTZ-B	1.802	0.803	0.284	
Regular dentifrice	HTZ-A	8.722	0.394	0.139	0.313
	HTZ-B	8.346	0.937	0.331	
Whitening dentifrice	HTZ-A	6.349	1.248	0.441	**0.0001**
	HTZ-B	11.062	0.387	0.137	

SD: standard deviation; SE: standard error.

Bold values show statistical significance

In addition to ΔE₀₀, directional changes in L*, a* and b* values were recorded. The main findings are summarised here:

1. HTZ-A:
  ○ ΔL*: Slight negative changes (−1.75 to −2.10) were observed with dentifrice exposure, indicating surface darkening.
  ○ Δa*: Positive shifts (+ 0.28 to + 0.35) indicate a redder hue, likely due to minor surface alterations.
  ○ Δb*: Positive shifts (+ 0.95 to + 1.25) indicate yellowing, attributable to abrasive effects of the dentifrice.
2. HTZ-B:

○ ΔL*: Greater negative changes (−2.00 to −2.45), reflecting more pronounced darkening, particularly with whitening dentifrice.
○ Δa*: Positive shifts (+ 0.40 to + 0.50) indicate a red hue shift, consistent with surface micro-abrasion.
○ Δb*: Positive shifts (+ 1.15 to + 1.60) indicate yellowing, most pronounced with whitening dentifrice due to higher abrasivity and chemical effects.

Both HTZ-A and HTZ-B exhibited slight darkening (ΔL < 0)* after exposure to dentifrices, with the largest effect observed in the whitening dentifrice group. Positive shifts in a* and b* were observed, indicating a trend towards redder and more yellowish hues, particularly in HTZ-B. Specimens stored in artificial saliva showed minimal changes in all three parameters.

## Discussion

Colour stability is a critical parameter for the long-term success of aesthetic restorations, influencing both patient satisfaction and clinical outcomes. The present study evaluated the influence of simulated toothbrushing using regular and whitening dentifrices on the colour stability (ΔE) of two HTZ materials with different yttria contents. In this study, dentifrice type significantly affected the ΔE values of HTZ samples. For HTZ-A, brushing with regular dentifrice produced the highest ΔE (8.722), followed by whitening dentifrice (6.348), whereas artificial saliva resulted in minimal change (1.677). In HTZ-B, whitening dentifrice induced the largest colour change (11.062), followed by regular dentifrice (8.346) and artificial saliva (1.802).

Two dentifrices, a regular dentifrice with low abrasiveness and a whitening dentifrice with high abrasiveness, were employed in the present study. These dentifrices were found to have RDA values of 70 and 100, respectively. The above-mentioned results summarise that whitening dentifrice has increased abrasive potential and causes alterations in surface and optical properties.

The differential colour change between HTZ-A and HTZ-B can be attributed to differences in yttria content and phase composition. HTZ-A, with higher cubic phase, exhibits greater optical sensitivity, making it more susceptible to colour alteration under low-abrasive conditions such as regular dentifrice. In contrast, HTZ-B, with higher tetragonal content, demonstrates increased resistance to mild abrasion but is more prone to surface degradation under highly abrasive whitening dentifrice, possibly due to microstructural damage and stress-induced phase transformation. Additionally, the combined abrasive and chemical action of whitening agents further accentuate surface roughness and optical changes in HTZ-B.

As a statistically significant difference was observed between the two major groups with respect to simulated tooth brushing using whitening dentifrice, the null hypothesis was rejected. These findings align with previous research reporting that dentifrices with higher RDA values exert a more pronounced effect on the surface integrity and colour stability of dental materials [12–16].

Lee et al. [17] reported statistically significant shade change in glazed zirconia specimens after 17 years of simulated brushing with whitening dentifrice [17]. Studies conducted by Roopa et al. [18] and Celik and Iscan Yapar [19] also observed substantial colour changes in aesthetic restorative materials following exposure to whitening dentifrices [18, 19]. The authors attributed these changes to the active ingredients of whitening formulations, notably hydrated silica and hydrogen peroxide. The major ingredient of whitening dentifrice employed in the present study contains hydrated silica and hydrogen peroxide. Hydrated silica acts as an abrasive agent, whereas hydrogen peroxide aids in stain removal. Hydrogen peroxide chemically interact with the chromophores of tooth tissues by breaking down their molecules and changing their size, geometry and polarity to alter the colour of the teeth. The results obtained are in accordance with the previous studies stating that whitening dentifrice exhibited higher colour change of HTZ-B.

However, some previous studies reported contrasting outcomes. Investigations by Bativala et al. [20], Sehovic [21] and Mahrous et al. [22] concluded that toothbrushing had minimal or no significant effect on surface roughness and colour stability of restorative materials [20–22]. These discrepancies may be attributed to differences in material composition, dentifrice abrasivity, surface finishing techniques or methodological variations and showed only a slight change in colour, which was not statistically significant.

The study underscores the importance of patient education regarding dentifrice selection, especially for individuals with highly translucent zirconia restorations. Use of low-abrasive or enamel-safe whitening agents may minimise colour change. Additionally, manufacturers may consider optimising surface hardness and chemical resistance to enhance the long-term aesthetic performance ceramic materials.

The main limitation of this study is its in vitro design, which may not accurately replicate intraoral conditions such as fluctuating pH, salivary composition, enzymatic activity, temperature changes or masticatory forces. Additionally, the flat, uniform specimen surfaces used in this study differ from the complex contours of clinical restorations, which may influence the abrasivity and optical outcomes.

Future research should include longitudinal clinical studies to evaluate real-life effects on colour stability with multiple simulated time points, considering dietary and oral hygiene variations. Investigating different polishing protocols, glazing techniques and low-abrasive whitening agents may provide guidance for optimising long-term aesthetic outcomes for high translucent zirconia restorations.

## Conclusion

Within the limitations of this in vitro study, simulated toothbrushing with whitening and regular dentifrices significantly affected the colour stability of both HTZ materials tested. Whitening dentifrice, characterised by higher abrasivity, produced the greatest colour change, particularly in the zirconia material with higher yttria content. These findings highlight the influence of dentifrice abrasiveness on the surface and optical properties of HTZ restorations and underscore the need for careful dentifrice selection to preserve long-term aesthetics.

## Data Availability

Within the article or its supplementary materials.
